# Stable isotope signatures of pelotons and rhizomorphs reveal nutritional plasticity in *Cremastra appendiculata* (Orchidaceae)

**DOI:** 10.1007/s00572-026-01315-x

**Published:** 2026-10-06

**Authors:** Kenji Suetsugu, Hidehito Okada, Akihiro Nishimura

**Affiliations:** 1 Department of Biology, Graduate School of Science, Kobe University, Kobe, 657-8501 Hyogo Japan; 2 Institute for Advanced Research, Kobe University, 1-1 Rokkodai, Nada-ku, Kobe, 657-8501 Hyogo Japan

**Keywords:** Coralloid rhizome, Mycoheterotrophy, Mycorrhiza, Peloton, Psathyrellaceae, Stable isotopes

## Abstract

Many photosynthetic orchids combine photosynthesis with fungal carbon acquisition, yet the isotopic relationships between orchids and their fungal partners remain poorly resolved. Direct stable isotope analysis of pelotons and extraradical fungal tissues can refine fungal baselines and clarify nutritional dynamics in orchid mycorrhizas. We investigated *Cremastra appendiculata*, a photosynthetic orchid with contrasting mycorrhizal states, by comparing fungal communities between individuals with and without coralloid rhizomes and analyzing the δ^13^C and δ^15^N values of leaves, protocorms, coralloid rhizomes, pelotons, Psathyrellaceae rhizomorphs, dead wood, and autotrophic reference plants. Psathyrellaceae-associated individuals were strongly enriched in ^13^C relative to autotrophic reference plants, indicating substantial acquisition of fungal carbon, whereas rhizoctonia-associated individuals exhibited little ^13^C enrichment and appeared predominantly autotrophic. Leaf δ^15^N values exceeded those of the associated fungal tissues in both states, suggesting plant-mediated ^15^N enrichment during nitrogen assimilation and allocation to leaves. Coralloid rhizome pelotons did not differ detectably in δ^13^C or δ^15^N between digestion stages. Pelotons resembled extraradical Psathyrellaceae rhizomorphs in δ^15^N but had higher δ^13^C values, indicating tissue-specific carbon isotope baselines. Psathyrellaceae tissues were less ^13^C enriched than fungal tissues reported from fully mycoheterotrophic orchids associated with wood-decaying fungi. These results support contrasting nutritional states in *C. appendiculata*: a predominantly autotrophic, rhizoctonia-associated state and a more fungus-dependent, Psathyrellaceae-associated state. The fungal isotope pattern raises the possibility that bidirectional carbon transfer persists even in the latter state. Determining whether the orchid transfers carbon to its fungal partners requires controlled culture or tracer experiments.

**Supplementary Information:**

The online version contains supplementary material available at https://doi.org/10.1007/s00572-026-01315-x.

## Introduction

With more than 31,000 accepted species, Orchidaceae is among the most species-rich plant families (Govaerts 2026). Because orchid seeds are minute and contain limited nutrient reserves, orchids depend on fungal symbionts for carbon (C), nitrogen (N), and other resources during germination and early development (Rasmussen 2002; Dearnaley et al. 2012). More than 200 orchid species remain fully mycoheterotrophic throughout their life cycles, obtaining all their carbon from fungi rather than through photosynthesis (Merckx 2013). Many photosynthetic orchids also acquire fungal carbon to varying degrees, a nutritional mode termed partial mycoheterotrophy (Gebauer and Meyer 2003; Bidartondo et al. 2004; Julou et al. 2005; Gebauer et al. 2016).

Most photosynthetic orchids associate with rhizoctonia fungi, including members of Serendipitaceae, Ceratobasidiaceae, and Tulasnellaceae (Rasmussen 2002; Dearnaley et al. 2012). These fungi may function as saprotrophs, endophytes, or pathogens and, less commonly, as ectomycorrhizal fungi, although their ecological roles remain incompletely understood in many systems (Dearnaley et al. 2012; Selosse and Martos 2014). In contrast, fully mycoheterotrophic orchids predominantly associate with ectomycorrhizal fungi or saprotrophic non-rhizoctonia fungi, which may provide access to larger pools of fungus-derived carbon (Hynson et al. 2013; Jacquemyn and Merckx 2019; Ogura-Tsujita et al. 2021).

Stable isotope analysis provides a powerful means of assessing fungal resource use in orchids. Fully mycoheterotrophic orchids generally exhibit higher δ^13^C, δ^15^N, and δ^2^H values than neighboring autotrophic C_3_ plants, reflecting the isotopic signatures of their fungal partners and subsequent fractionation during transfer and assimilation (Gebauer and Meyer 2003; Trudell et al. 2003; Gebauer et al. 2016). Some photosynthetic orchids exhibit intermediate isotopic profiles, consistent with partial mycoheterotrophy (Gebauer and Meyer 2003; Bidartondo et al. 2004; Julou et al. 2005). Isotope patterns can also help distinguish fungal nutritional guilds: orchids associated with ectomycorrhizal fungi often show stronger ^15^N enrichment, whereas those associated with saprotrophic fungi, particularly wood-decaying fungi, often exhibit stronger ^13^C enrichment (Martos et al. 2009; Ogura-Tsujita et al. 2009; Lee et al. 2015; Suetsugu et al. 2020). δ^13^C values can also be incorporated into two-source mixing models to estimate the contribution of fungus-derived carbon to green orchids when suitable isotopic endpoints are available (Gebauer and Meyer 2003; Hynson et al. 2013).

A central methodological challenge is defining isotope baselines for the fungal partners themselves. Recent studies have addressed this issue by analyzing fungal hyphae or pelotons (hyphal coils) extracted directly from orchid tissues (Gomes et al. 2023; Zahn et al. 2023). This approach is particularly useful for rhizoctonia fungi and other orchid mycorrhizal fungi that rarely produce conspicuous fruiting bodies. However, peloton extraction also raises methodological concerns. Digested pelotons could differ isotopically from intact fungal tissues if lysis selectively removes ^15^N-enriched cytoplasmic contents while retaining ^15^N-depleted structural material (Taylor et al. 1997; Hobbie and Colpaert 2003; Gomes et al. 2023; Zahn et al. 2023). Direct comparisons among pelotons at different digestion stages and extraradical fungal tissues are therefore needed to determine when pelotons provide reliable fungal isotopic baselines.

The photosynthetic orchid *Cremastra appendiculata* provides a useful model for investigating contrasting mycorrhizal and nutritional states within a single species. Individuals bearing coralloid rhizomes obtain approximately 50% or more of their carbon from decomposing wood through Psathyrellaceae fungi (Suetsugu et al. 2022). Stable isotope analysis has revealed strong ^13^C enrichment in these individuals relative to autotrophic reference plants, while radiocarbon data demonstrate acquisition of decades-old carbon derived from decomposing wood (Suetsugu et al. 2022). In contrast, individuals lacking coralloid rhizomes associate primarily with rhizoctonia fungi and exhibit δ^13^C and δ^15^N values similar to those of autotrophic reference plants (Yagame et al. 2021). Zahn et al. (2022) further demonstrated marked ontogenetic plasticity: protocorms and seedlings bearing coralloid rhizomes depend on Psathyrellaceae fungi, whereas mature individuals lacking these structures exhibit no detectable ^13^C, ^15^N, or ^2^H enrichment. Although rhizoctonia-associated orchids may exhibit limited ^13^C enrichment even when fungal carbon is acquired (Gomes et al. 2023; Zahn et al. 2023), the absence of ^2^H enrichment provides strong evidence for predominantly autotrophic nutrition in this state (Zahn et al. 2022).

Moreover, *C. appendiculata* is particularly well suited for evaluating peloton isotopic baselines in Psathyrellaceae-associated partial mycoheterotrophy. Its large coralloid rhizomes contain largely intact fungal hyphae in outer tissues and more extensively digested pelotons in inner tissues. This separation allows weakly and strongly digested pelotons to be compared. In addition, Psathyrellaceae fungi commonly form rhizomorphs, allowing direct comparisons between intraradical pelotons and extraradical fungal tissues. We therefore focused on this species for an integrated stable isotope analysis encompassing both pelotons and intact rhizomorphs.

After confirming that *C. appendiculata* individuals with and without coralloid rhizomes harbored distinct mycorrhizal communities, we measured the δ^13^C and δ^15^N values of leaves, protocorms, coralloid rhizomes, root-derived pelotons, weakly and strongly digested coralloid rhizome pelotons, Psathyrellaceae rhizomorphs, dead wood, and autotrophic reference plants. This design allowed us to assess population-level variation in dependence on fungal carbon, test whether peloton digestion biases fungal isotopic baselines in Psathyrellaceae-associated tissues, and characterize isotopic relationships between plant and fungal tissues across contrasting mycorrhizal states.

## Materials and methods

### Study site and sampling scheme

Fieldwork was conducted on December 4, 2023, in a population of *C. appendiculata* in Hara, Fujinomiya City, Shizuoka Prefecture, Japan. The site was a warm-temperate forest dominated by *Cryptomeria japonica*, *Morus australis*, and *Zelkova serrata*. Approximately 100 individuals of *C. appendiculata* occurred at the site. Individuals bearing coralloid rhizomes occurred predominantly near decaying logs and beneath the litter layer.

We established five 1 × 1 m plots, each containing *C. appendiculata* individuals with and without coralloid rhizomes. When multiple orchid individuals were sampled within a plot, they were selected as far apart as practicable. Within these plots, we collected leaves and mycorrhizal tissues for stable isotope analysis, peloton extraction, and molecular identification of mycorrhizal fungi. Tissues obtained from the same orchid were assigned a common individual identifier, which was also used for the corresponding molecular samples (Tables S1, S2). In addition, dead wood, Psathyrellaceae rhizomorphs associated with *C. appendiculata* coralloid rhizomes, and leaves from three to five co-occurring autotrophic plant species were collected from each plot following Gebauer and Meyer (2003). *Cremastra appendiculata* protocorms were included when encountered beneath decaying wood: seven were analyzed for stable isotopes, and six of these were also used for molecular analysis. Intact roots from individuals lacking coralloid rhizomes were not analyzed as a separate isotopic category because the limited material was prioritized for peloton extraction and molecular identification.

**Fig. 1:**
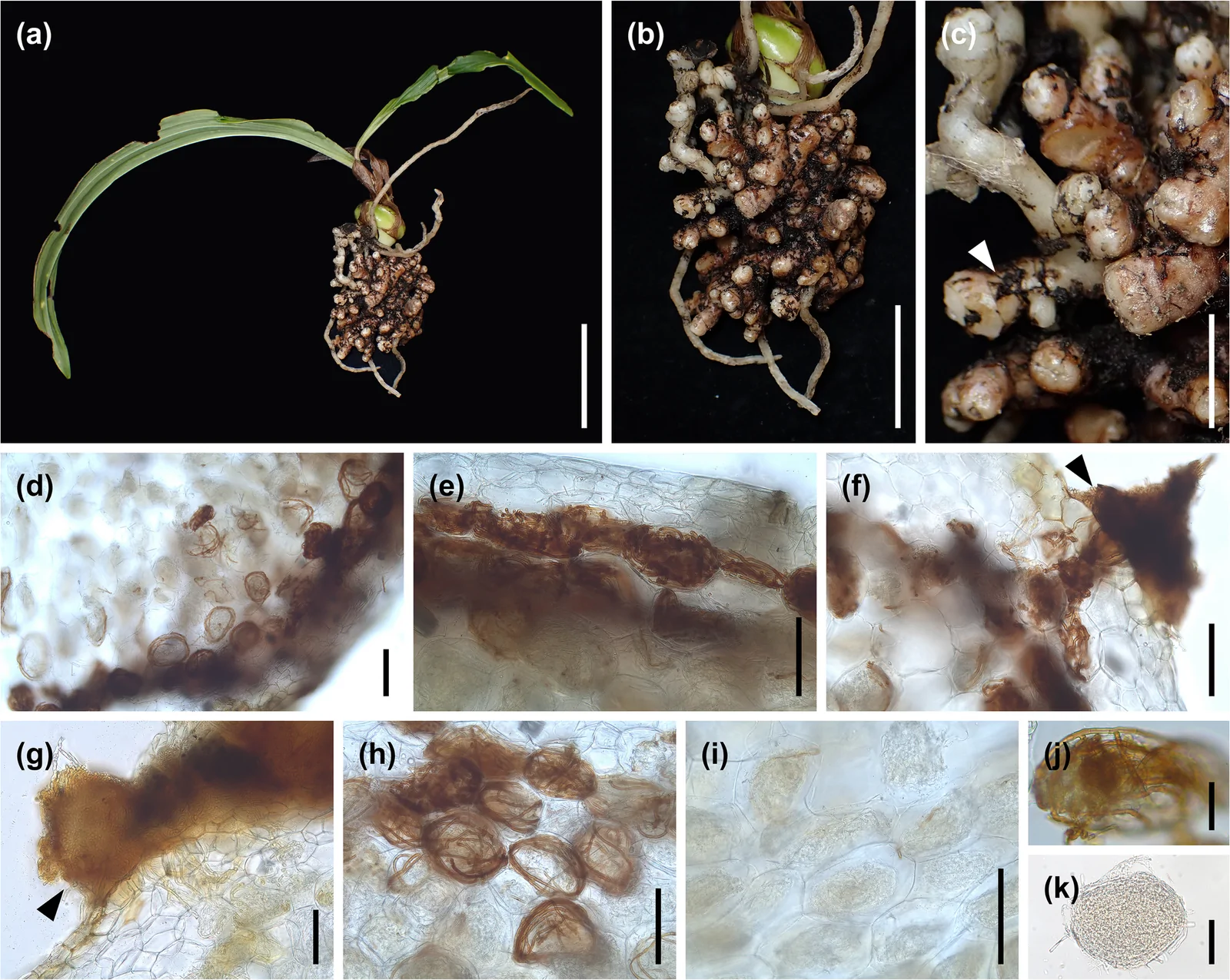
Mycorrhizal association between *Cremastra appendiculata* individuals bearing coralloid rhizomes and Psathyrellaceae fungi. **a** Whole plant bearing a coralloid rhizome. **b** Subterranean organs, including the corm, roots, and coralloid rhizome. **c** Enlarged view of the coralloid rhizome and an associated extraradical rhizomorph (arrowhead). **d**,** e** Tissue sections showing fungal colonization in the outer and inner regions of a coralloid rhizome. **f**,** g** Connections between extraradical rhizomorphs (arrowheads) and fungal hyphae entering the coralloid rhizome. **h** Coralloid rhizome cells containing weakly digested pelotons. **i** Coralloid rhizome cells containing strongly digested pelotons. **j** Weakly digested peloton extracted from a coralloid rhizome cell. **k** Strongly digested peloton extracted from a coralloid rhizome cell. Scale bars: 5 cm (**a**), 3 cm (**b**), 1 cm (**c**), 100 μm (**d–i**), and 30 μm (**j**,** k**)

**Fig. 2:**
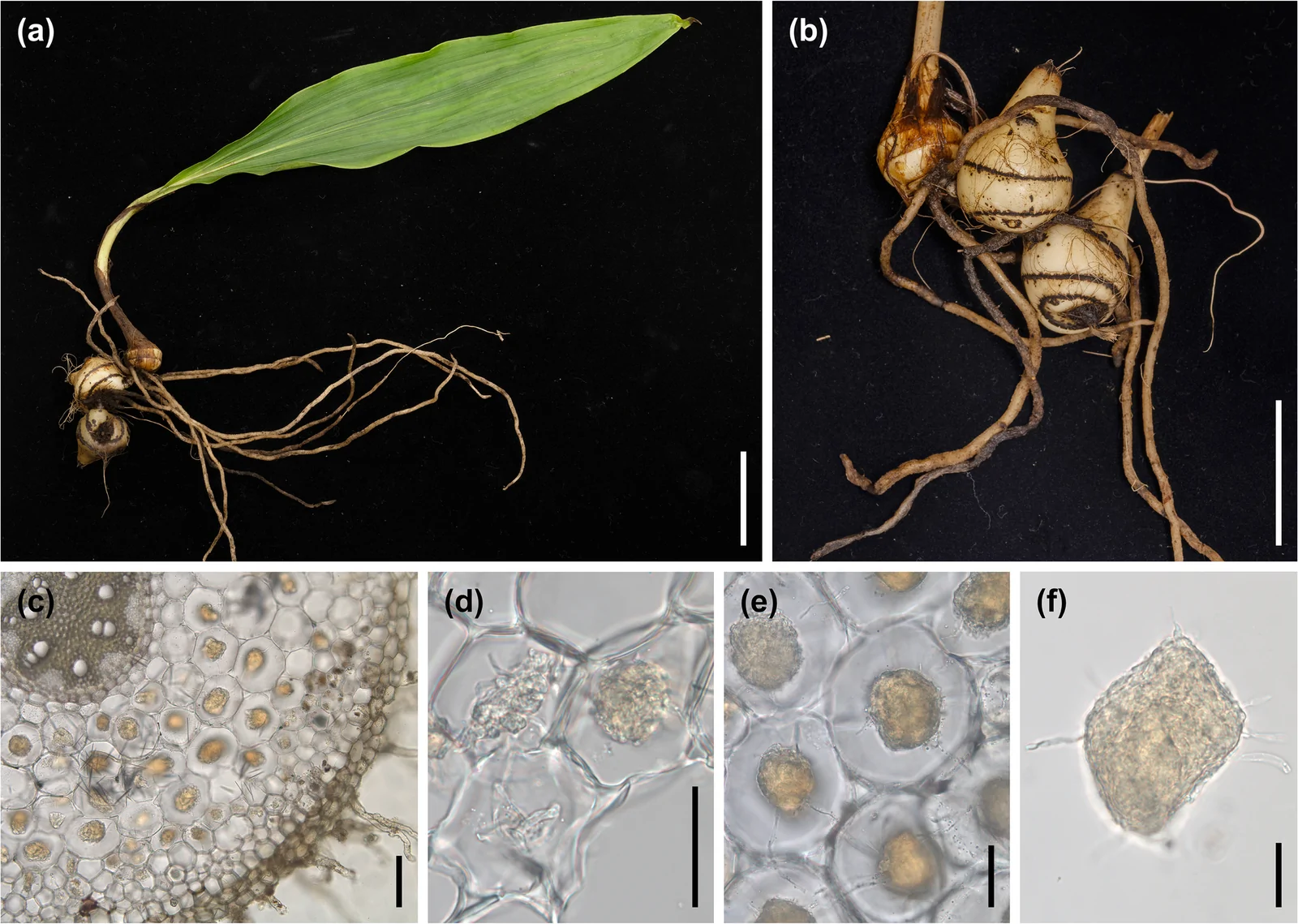
Mycorrhizal association between *Cremastra appendiculata* individuals lacking coralloid rhizomes and rhizoctonia fungi. **a** Whole plant lacking a coralloid rhizome. **b** Subterranean organs, including the corms and roots. **c** Root cross-section showing colonized cortical cells. **d** Root cortical cells containing relatively intact pelotons. **e** Root cortical cells containing more extensively digested pelotons. **f** Peloton extracted from a root cortical cell. Scale bars: 5 cm (**a**), 3 cm (**b**), 300 μm (**c**), 100 μm (**d**,** e**), and 30 μm (**f**)

Fungal colonization within coralloid rhizomes was spatially structured, with largely intact hyphae in outer tissues and more extensively digested pelotons in inner tissues. Accordingly, coralloid rhizome pelotons were classified as weakly or strongly digested (Fig. 1). We therefore separated the outer and inner tissues of each coralloid rhizome and processed them independently to obtain weakly and strongly digested peloton samples, respectively (Fig. 1). In contrast, root-derived pelotons were pooled across digestion stages within each individual because material was limited and spatial differentiation was less distinct (Fig. 2). Pelotons were extracted and purified following Suetsugu and Okada (2025a). Briefly, mycorrhizal tissues were cut longitudinally with a razor blade and sonicated in deionized water for 15 min. The resulting suspension was transferred to a separate tube, and extraction with fresh water was repeated until the suspension became clear. The pooled suspensions were filtered through stacked stainless-steel sieves with mesh sizes of 250, 106, and 53 µm (Sanpo, Tokyo, Japan). After thorough rinsing with deionized water, pelotons were recovered mainly from the 106-µm sieve, transferred to microcentrifuge tubes, and centrifuged for 10 s before the supernatant was discarded.

### Molecular identification of mycorrhizal fungi

Mycorrhizal tissue fragments (3–5 mm) containing fungal pelotons were collected from *C. appendiculata* individuals bearing coralloid rhizomes, individuals lacking coralloid rhizomes, and protocorms (*n* = 7, 5, and 6, respectively). Rhizome-bearing individuals were sampled across all five plots (2, 2, 1, 1, and 1 individuals from plots 1 through 5, respectively), and one rhizome-lacking individual was sampled per plot. Protocorm samples came from plots 1 through 4 (2, 1, 2, and 1 samples, respectively; Table S1). The samples were surface sterilized, and DNA was extracted using the cetyltrimethylammonium bromide method (Doyle and Doyle 1990).

We amplified the nuclear ribosomal internal transcribed spacer (ITS) region using two primer sets: ITS86F/ITS4, which is widely used to characterize fungal communities in orchid roots (Waud et al. 2014), and a *Tulasnella*-specific set. *Tulasnella* can be strongly underestimated by universal fungal primers (Rammitsu et al. 2021). The published primer pair 5.8 S-Tulngs/ITS4-Tul2 also performed poorly in preliminary tests. We therefore designed the forward primer 5.8 S-Tulngs-7 (5′-CCTTGGCACGTCATTC-3′) and paired it with the reverse primer ITS4-Tul2 (Oja et al. 2015). First-round primers carried Illumina sequencing-primer sequences and 3–6 random nucleotides to increase sequence diversity. PCRs used the Q5 High-Fidelity DNA Polymerase Kit (New England Biolabs, Ipswich, MA, USA) with initial denaturation at 98 °C for 40 s; 35 cycles of 98 °C for 5 s, 58 °C for 10 s for ITS86F/ITS4 or 61 °C for 10 s for 5.8 S-Tulngs-7/ITS4-Tul2, and 72 °C for 20 s; followed by a final extension at 72 °C for 10 min. A second PCR added Illumina P5 and P7 adapters and sample-specific indices (Syed et al. 2009). Cycling comprised initial denaturation at 98 °C for 40 s; 12 cycles of 98 °C for 5 s, 65 °C for 10 s, and 72 °C for 20 s; and a final extension at 72 °C for 10 min. Amplicon libraries were sequenced using either an Element AVITI system with the AVITI 2 × 150 Sequencing Kit Cloudbreak FS Low Output (Element Biosciences, San Diego, CA, USA) or an Illumina MiSeq system with the MiSeq Reagent Micro Kit v2 (300 cycles; Illumina, San Diego, CA, USA). Because our ecological conclusions were based on dominant OTUs and broad community contrasts rather than variation among low-abundance taxa, potential platform effects were unlikely to alter the main interpretations. All sequence data were deposited in the NCBI Sequence Read Archive under BioProject accession PRJNA1443979.

Sequences were processed using Claident v0.9.2025.09.29 (Tanabe and Toju 2013). Primer regions and low-quality reads were removed, and sequences suspected to be PCR chimeras or index-hopping artifacts were excluded using clremovechimev and clremovecontam (Esling et al. 2015; Nilsson et al. 2019). The remaining reads were clustered into operational taxonomic units (OTUs) at 97% similarity using VSEARCH v2.8.0 (Rognes et al. 2016). The most abundant sequence in each OTU was selected as its representative. Taxonomic assignments were made using the query-centric auto-k-nearest-neighbor (QCauto) and lowest common ancestor (LCA) algorithms (Huson et al. 2007) with the Claident “overall_genus” reference database. To minimize the effects of rare OTUs and unequal sequencing depths, we first removed OTUs represented by a single read within an individual PCR amplicon or by ≤ 10 reads across the complete data matrix. Each sample was then rarefied to 4,954 reads.

Representative sequences of the dominant OTUs were queried against the International Nucleotide Sequence Database Collaboration (INSDC) databases using BLAST (Altschul et al. 1997), and closely related sequences were retrieved. Sequences were aligned with PRANK v170427 (Löytynoja 2021), and maximum-likelihood trees were inferred with IQ-TREE v3.0.1 (Wong et al. 2026). The best-fitting substitution model was selected by ModelFinder according to the Bayesian information criterion (Kalyaanamoorthy et al. 2017). Branch support was evaluated using 1,000 replicates each of the Shimodaira–Hasegawa-like approximate likelihood-ratio test (SH-aLRT; Guindon et al. 2010) and ultrafast bootstrap analysis (UFBoot; Hoang et al. 2018).

### Stable isotope analysis

We analyzed 70 samples from the five plots, comprising leaves from *C. appendiculata* individuals bearing coralloid rhizomes (*n* = 6) and lacking coralloid rhizomes (*n* = 5), coralloid rhizomes (*n* = 6), weakly digested pelotons from coralloid rhizomes (*n* = 6), strongly digested pelotons from coralloid rhizomes (*n* = 6), pelotons from individuals lacking coralloid rhizomes (*n* = 5), dead wood samples (*n* = 5), protocorms (*n* = 7), Psathyrellaceae rhizomorphs (*n* = 6), and leaves from autotrophic reference plants (*n* = 18). Samples were dried at 60 °C and ground with an agate mortar. The natural abundances of ^13^C and ^15^N were measured using a continuous-flow isotope-ratio mass spectrometer coupled to an elemental analyzer (Thermo Fisher Scientific, Waltham, MA, USA). Relative isotope abundances were calculated as δ^13^C or δ^15^N = (*R*_sample_/*R*_standard_ – 1) × 1,000 [‰], where *R*_sample_ represents the ^13^C/^12^C or ^15^ N/^14^N ratio of the sample, and *R*_standard_ represents the corresponding ratio of Vienna Pee Dee Belemnite (VPDB) or atmospheric N_2_, respectively. Calibration was performed using the laboratory reference materials CERKU-01, CERKU-02, and CERKU-03 (Tayasu et al. 2011). Analytical precision was < 0.09‰ for δ^13^C (*n* = 24) and < 0.22‰ for δ^15^N (*n* = 24). Carbon and nitrogen concentrations were determined from the CO_2_ and N_2_ peak areas relative to sample mass (Tayasu et al. 2011).

To facilitate comparisons with previous studies, enrichment factors (ε) were calculated as ε = δ_S_ − δ_REF_, where δ_S_ is the δ^13^C or δ^15^N value of the target sample, including *C. appendiculata* tissues, associated fungal tissues, and dead wood, and δ_REF_ is the mean δ^13^C or δ^15^N value of autotrophic reference plants from the same plot (Preiss and Gebauer 2008). The proportion of fungus-derived carbon (% C_df_) was estimated using a linear two-source mixing model: % C_df_ = (ε^13^C_sample_/ε^13^C_endpoint_) × 100. Local autotrophic reference plants (ε^13^C = 0‰) represented the 0% fungal carbon endpoint, whereas the mean ε^13^C value of the relevant nonphotosynthetic plant or fungal tissue served as an operational 100% endpoint. For individuals bearing coralloid rhizomes, estimates were calculated separately using either protocorms or the pooled mean of weakly and strongly digested coralloid rhizome pelotons as the endpoint. Root pelotons were used for individuals lacking coralloid rhizomes.

### Statistical analyses

Differences in δ^13^C, δ^15^N, and nitrogen concentration among the 10 sample categories were evaluated using linear mixed-effects models fitted with the lmer function in *lme4* v2.0-1 (Bates et al. 2015). Sample category was included as a fixed effect, whereas plot and biological sampling unit were included as random intercepts to account for clustering within plots and repeated tissues from the same orchid, respectively. Tissues from the same *C. appendiculata* individual and its associated rhizomorphs shared a common biological sampling-unit identifier, whereas each protocorm, dead wood sample, and autotrophic reference plant was assigned a unique identifier. Pairwise comparisons were conducted using the glht function in *multcomp* v1.4-30 (Hothorn et al. 2008), with Tukey contrasts and single-step multiplicity adjustment across all 45 comparisons for each response; *z* statistics were reported for individual contrasts. All analyses were performed in R v4.6.0 (R Core Team 2026).

## Results

### Molecular identification of mycorrhizal fungi

**Fig. 3:**
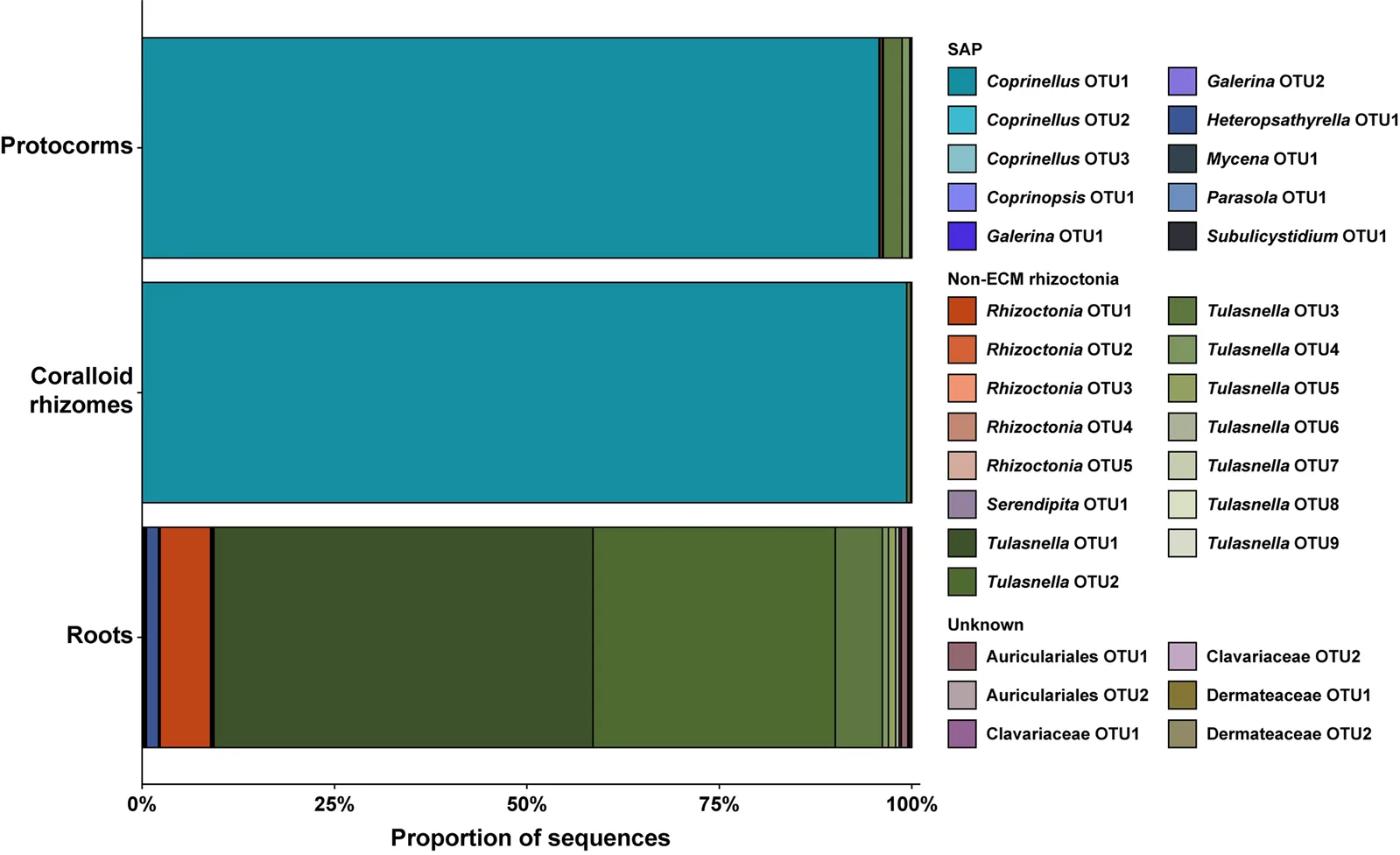
Relative read composition of fungal operational taxonomic units detected in *Cremastra appendiculata* protocorms, coralloid rhizomes, and roots from individuals lacking coralloid rhizomes. Bars show read proportions pooled within each sample category after filtering and rarefaction. Operational taxonomic units are grouped as saprotrophic fungi (SAP), non-ectomycorrhizal rhizoctonia fungi (non-ECM rhizoctonia), or fungi of unknown nutritional mode

Metabarcoding revealed a clear association between the coralloid rhizomes of *C. appendiculata* and wood-decaying Psathyrellaceae fungi. Across seven individuals bearing coralloid rhizomes, 34,678 reads were assigned to seven OTUs. *Coprinellus* OTU1 occurred in every individual and accounted for 34,447 reads (99.33%; Fig. 3; Table S1). The remaining OTUs collectively represented only 0.67% of the reads and were treated as minor components of the sequence dataset rather than major mycorrhizal associates (Table S1).

**Fig. 4:**
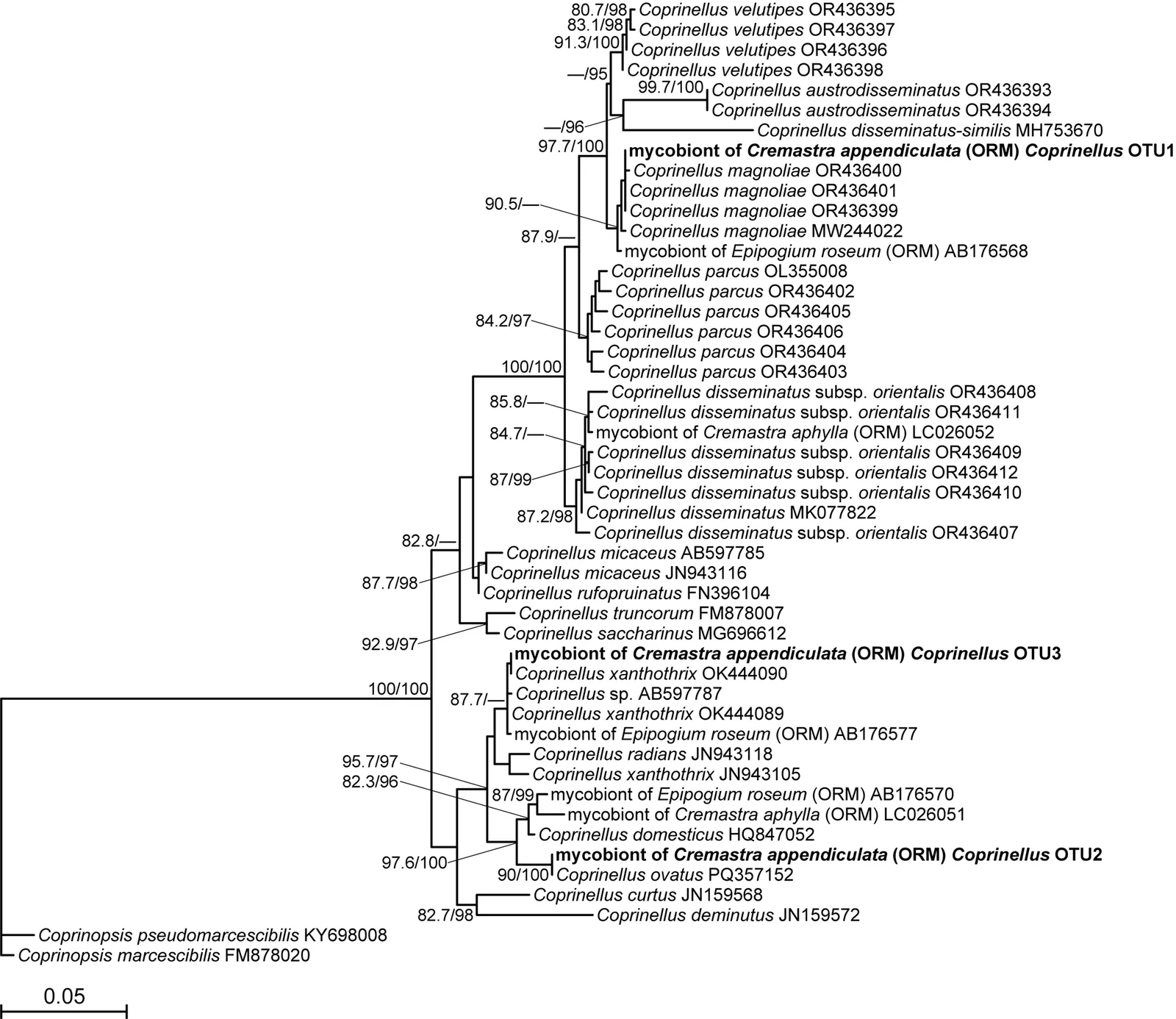
Maximum-likelihood phylogeny based on ITS2 sequences of *Coprinellus* operational taxonomic units detected in mycorrhizal samples of *Cremastra appendiculata* (bold) and reference sequences retrieved from the International Nucleotide Sequence Database Collaboration (INSDC). OTUs detected in *C. appendiculata* are numbered in decreasing order of read abundance; accession numbers are provided for reference sequences. The tree was rooted with *Coprinopsis pseudomarcescibilis* and *Coprinopsis marcescibilis* (Psathyrellaceae). Node labels give SH-aLRT/UFBoot support percentages; only SH-aLRT values ≥ 80% and UFBoot values ≥ 95% are shown, and dashes indicate values below the corresponding thresholds. Scale bar indicates substitutions per site. ORM, orchid mycorrhizal fungi

Protocorm fungal communities closely resembled those of coralloid rhizomes. Six protocorms yielded 29,724 reads assigned to 10 OTUs, again dominated by Psathyrellaceae. *Coprinellus* OTU1 occurred in all protocorms and accounted for 28,459 reads (95.74%); all other OTUs had low relative abundances (Table S1). Its sequence was identical to that previously recovered from a mycorrhizal rhizome of *C. aphylla*, the sister species of *C. appendiculata*, at a geographically distant site in Hachioji, Tokyo, Japan (Suetsugu et al. 2026). Phylogenetic analysis placed OTU1 close to *Coprinellus magnoliae* and mycorrhizal fungi previously isolated from *Epipogium roseum* (Fig. 4).

In contrast, *C. appendiculata* individuals lacking coralloid rhizomes harbored root-associated fungal communities dominated by typical orchid mycorrhizal fungi. Five root samples yielded 24,770 reads assigned to 29 OTUs, with Tulasnellaceae and Ceratobasidiaceae constituting the major components. Tulasnellaceae comprised nine OTUs and 22,113 reads (89.27%) and was dominated by *Tulasnella* OTU1 (12,191 reads; 49.22%; five individuals), *Tulasnella* OTU2 (7,802 reads; 31.50%; four individuals), and *Tulasnella* OTU3 (1,514 reads; 6.11%; five individuals). Ceratobasidiaceae comprised three OTUs and 1,683 reads (6.79%), primarily *Rhizoctonia* OTU1 (1,621 reads; 6.54%). *Galerina* comprised two OTUs detected in three root samples (100 reads; 0.40% of root reads), while *Serendipita* OTU1 occurred in one root sample (49 reads; 0.20% of root reads; Table S1).

**Fig. 5:**
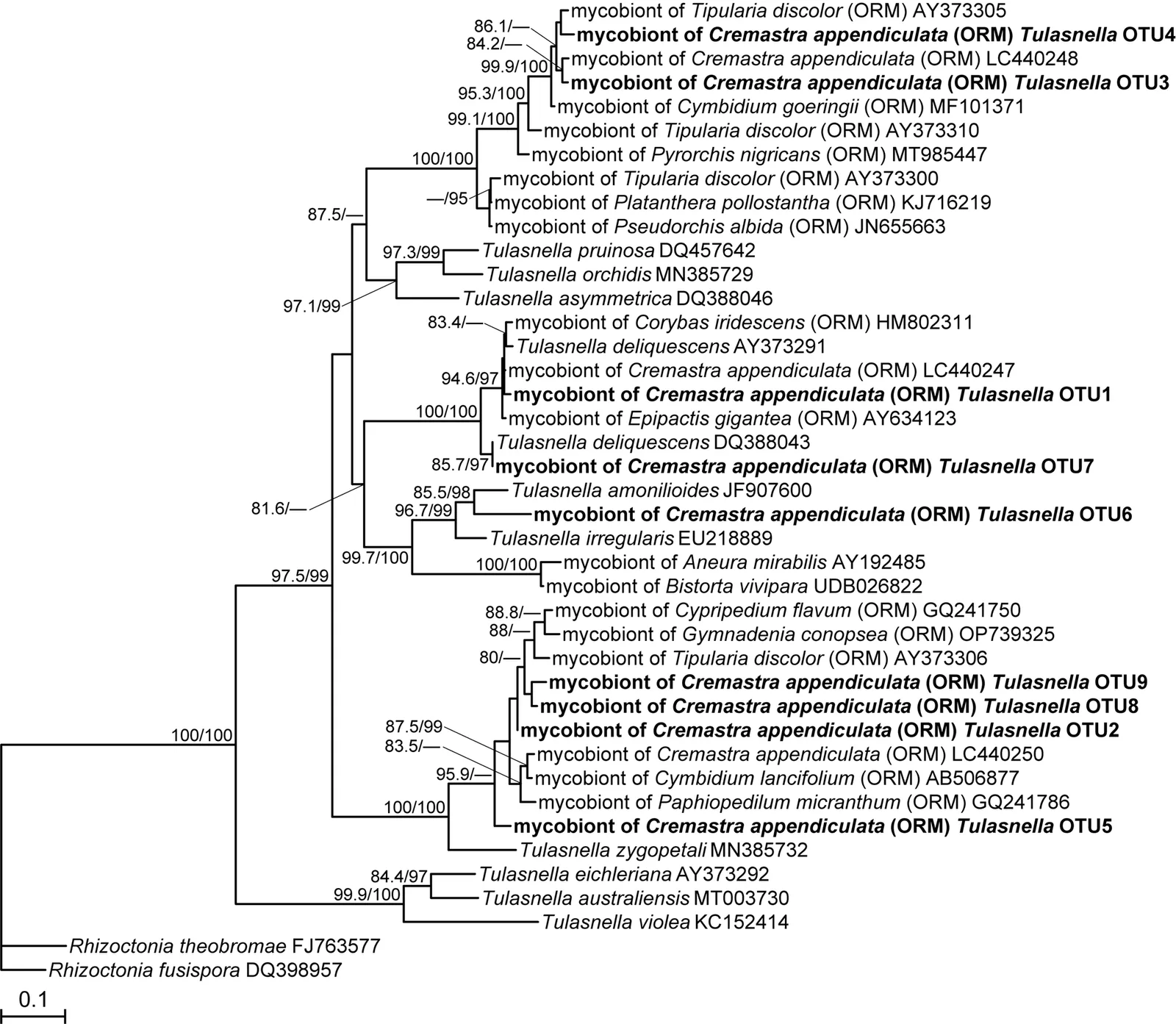
Maximum-likelihood phylogeny based on ITS2 sequences of Tulasnellaceae operational taxonomic units detected in mycorrhizal samples of *Cremastra appendiculata* (bold) and reference sequences retrieved from the International Nucleotide Sequence Database Collaboration (INSDC). OTUs detected in *C. appendiculata* are numbered in decreasing order of read abundance; accession numbers are provided for reference sequences. The tree was rooted with *Rhizoctonia fusispora* and *Rhizoctonia theobromae* (Ceratobasidiaceae). Node labels give SH-aLRT/UFBoot support percentages; only SH-aLRT values ≥ 80% and UFBoot values ≥ 95% are shown, and dashes indicate values below the corresponding thresholds. Scale bar indicates substitutions per site. ECM, ectomycorrhizal fungi; ORM, orchid mycorrhizal fungi

**Fig. 6:**
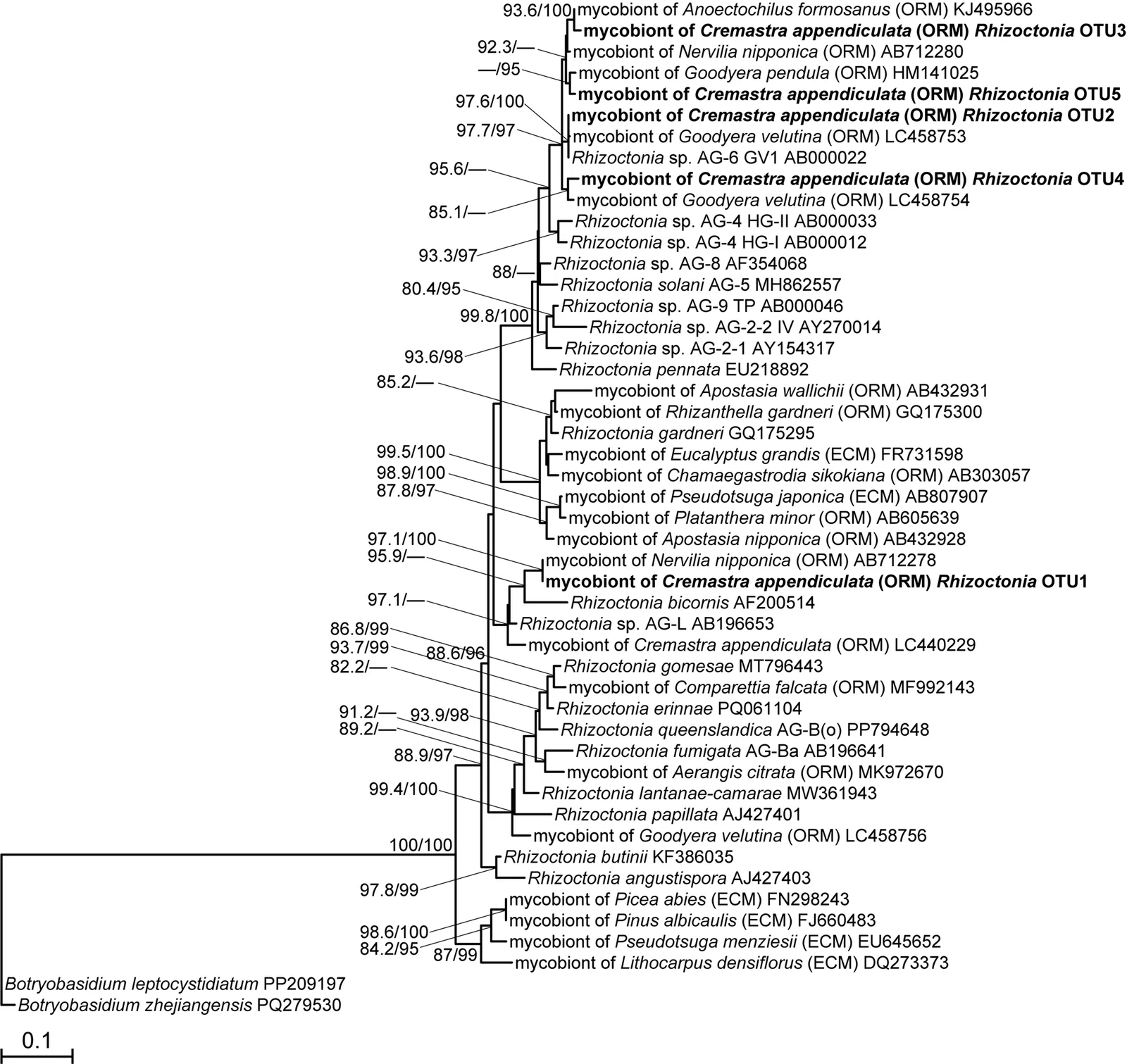
Maximum-likelihood phylogeny based on ITS2 sequences of Ceratobasidiaceae operational taxonomic units detected in mycorrhizal samples of *Cremastra appendiculata* (bold) and reference sequences retrieved from the International Nucleotide Sequence Database Collaboration (INSDC). OTUs detected in *C. appendiculata* are numbered in decreasing order of read abundance; accession numbers are provided for reference sequences. The tree was rooted with *Botryobasidium leptocystidiatum* and *Botryobasidium zhejiangensis* (Botryobasidiaceae). Node labels give SH-aLRT/UFBoot support percentages; only SH-aLRT values ≥ 80% and UFBoot values ≥ 95% are shown, and dashes indicate values below the corresponding thresholds. Scale bar indicates substitutions per site. ECM, ectomycorrhizal fungi; ORM, orchid mycorrhizal fungi

Phylogenetic analyses showed that most Tulasnellaceae and Ceratobasidiaceae OTUs detected in *C. appendiculata* were closely related to mycorrhizal fungi widely reported from rhizoctonia-associated orchids (Figs. 5 and 6). Although Ceratobasidiaceae contains two independently evolved ectomycorrhizal clades (Veldre et al. 2013), none of the Ceratobasidiaceae OTUs detected here belonged to either clade (Fig. 6).

### Stable isotope analysis

**Fig. 7:**
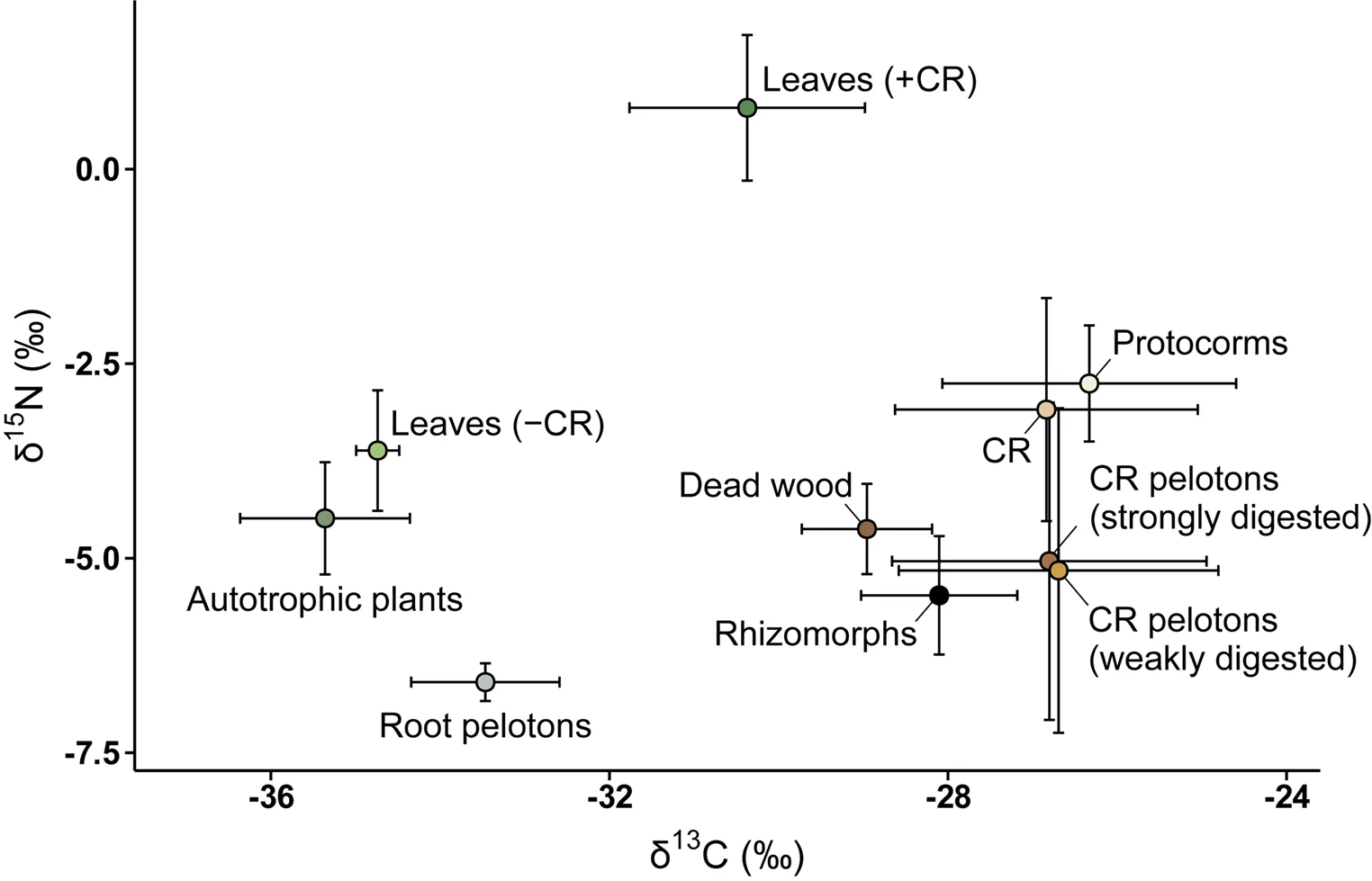
Mean (± SD) δ^13^C and δ^15^N values of *Cremastra appendiculata* tissues, associated fungal tissues, dead wood, and neighboring autotrophic reference plants. Sample sizes were seven protocorms; six coralloid rhizomes; six leaves from individuals bearing coralloid rhizomes; five leaves from individuals lacking coralloid rhizomes; six weakly digested and six strongly digested coralloid rhizome peloton samples; six Psathyrellaceae rhizomorph samples; five root peloton samples; five dead wood samples; and 18 autotrophic reference plant samples. CR, coralloid rhizomes; Leaves (+ CR), leaves of individuals bearing coralloid rhizomes; Leaves (− CR), leaves of individuals lacking coralloid rhizomes; Rhizomorphs, Psathyrellaceae rhizomorphs associated with coralloid rhizomes; Root pelotons, pelotons extracted from roots of individuals lacking coralloid rhizomes; CR pelotons (weakly digested) and CR pelotons (strongly digested), weakly and strongly digested pelotons extracted from coralloid rhizomes, respectively

The δ^13^C values of protocorms (− 26.3 ± 1.7‰; mean ± SD), coralloid rhizomes (− 26.8 ± 1.8‰), and leaves from *C. appendiculata* individuals bearing coralloid rhizomes (− 30.4 ± 1.4‰) were significantly higher than those of autotrophic reference plants (− 35.4 ± 1.0‰; linear mixed-effects models with Tukey-adjusted pairwise comparisons: *z* = 16.23, 15.20, and 9.12, respectively; all *P* < 0.001; Fig. 7).

The δ^13^C values of strongly digested pelotons (− 26.8 ± 1.9‰), weakly digested pelotons (− 26.7 ± 1.9‰), and Psathyrellaceae rhizomorphs associated with coralloid rhizomes (− 28.1 ± 0.9‰) were significantly higher than those of autotrophic reference plants (*z* = 15.34, 15.53, and 13.07, respectively; all *P* < 0.001). No significant difference in δ^13^C was detected between strongly and weakly digested pelotons (*z* = − 0.28, *P* = 1.000). However, weakly and strongly digested pelotons had higher δ^13^C values than rhizomorphs (*z* = 3.56, *P* = 0.012 and *z* = 3.28, *P* = 0.029, respectively). Dead wood samples that likely supported the Psathyrellaceae fungi had δ^13^C values (− 29.0 ± 0.8‰) that were higher than those of autotrophic reference plants (*z* = 10.14, *P* < 0.001). These values were lower than those of weakly and strongly digested pelotons (*z* = − 3.36, *P* = 0.023 and *z* = − 3.22, *P* = 0.036, respectively), but did not differ significantly from those of rhizomorphs (*z* = − 1.47, *P* = 0.887; Fig. 7).

In contrast, the δ^13^C values of leaves from *C. appendiculata* individuals lacking coralloid rhizomes (− 34.7 ± 0.3‰) did not differ significantly from those of autotrophic reference plants (*z* = 0.98, *P* = 0.991). Pelotons extracted from these individuals (− 33.5 ± 0.9‰) exhibited slightly higher δ^13^C values than the autotrophic reference plants, although the difference was not significant (*z* = 3.00, *P* = 0.069).

Using protocorms (mean ε^13^C = 8.9 ± 1.8‰) or pooled weakly and strongly digested coralloid rhizome pelotons (8.7 ± 1.7‰) as operational 100% fungal-carbon endpoints, leaves of rhizome-bearing *C. appendiculata* were estimated to derive 56.2 ± 14.7% and 58.1 ± 15.2% of their carbon from fungi, respectively. The corresponding estimates for coralloid rhizomes were 94.7 ± 17.8% and 98.0 ± 18.4%. Using root pelotons from individuals lacking coralloid rhizomes as the endpoint (ε^13^C = 1.9 ± 0.9‰) yielded an estimated fungal contribution of 32.1 ± 27.5% to leaf carbon. Because this endpoint was weakly enriched and overlapped autotrophic reference values, the latter estimate was highly uncertain and should not be interpreted as a precise source proportion.

The δ^15^N values of protocorms (− 2.8 ± 0.7‰) and leaves from individuals bearing coralloid rhizomes (0.8 ± 0.9‰) were significantly higher than those of autotrophic reference plants (− 4.5 ± 0.7‰; *z* = 3.49, *P* = 0.017 for protocorms and *z* = 9.96, *P* < 0.001 for leaves). In contrast, the δ^15^N values of coralloid rhizomes (− 3.1 ± 1.4‰), strongly digested pelotons (− 5.0 ± 2.0‰), weakly digested pelotons (− 5.2 ± 2.1‰), Psathyrellaceae rhizomorphs (− 5.5 ± 0.8‰), and dead wood (− 4.6 ± 0.6‰) did not differ significantly from those of autotrophic reference plants (*z* = 2.88, − 1.11, − 1.34, − 1.95, and − 0.25, respectively; all *P* ≥ 0.109). Although the leaf δ^15^N values of individuals lacking coralloid rhizomes (− 3.6 ± 0.8‰) did not differ significantly from those of autotrophic reference plants (*z* = 1.54, *P* = 0.873), pelotons extracted from these individuals (− 6.6 ± 0.2‰) had significantly lower δ^15^N values than the autotrophic references (*z* = − 3.73, *P* = 0.007). In both rhizome-bearing and rhizome-lacking individuals, leaf δ^15^N values were significantly higher than those of the corresponding pelotons (rhizome-bearing leaves versus strongly and weakly digested pelotons: *z* = 9.66 and 9.86, respectively; rhizome-lacking leaves versus root pelotons: *z* = 4.51; all *P* < 0.001). The δ^15^N values of weakly and strongly digested pelotons did not differ significantly from those of rhizomorphs (*z* = 0.53 and 0.73, respectively; both *P* ≥ 0.999) or from each other (strongly versus weakly digested: *z* = 0.20, *P* = 1.000).

Among *C. appendiculata* tissues, nitrogen concentrations were highest in leaves of individuals bearing coralloid rhizomes (4.98 ± 0.45%), followed by leaves of individuals lacking coralloid rhizomes (4.08 ± 0.39%), protocorms (3.68 ± 1.49%), and coralloid rhizomes (3.01 ± 0.17%). Pelotons from coralloid rhizomes, whether strongly digested (2.68 ± 0.71%) or weakly digested (2.82 ± 0.41%), had lower nitrogen concentrations than host leaves (Tukey-adjusted pairwise comparisons: *z* = − 9.69 and − 9.11, respectively; both *P* < 0.001) but higher concentrations than the dead wood substrate (0.82 ± 0.28%; *z* = 3.68, *P* = 0.007 and z = 3.96, *P* = 0.003, respectively). Nitrogen concentrations in leaves of individuals bearing coralloid rhizomes were significantly higher than those of autotrophic reference plants (3.23 ± 0.85%; *z* = 4.51, *P* < 0.001), whereas those in individuals lacking coralloid rhizomes did not differ significantly from the autotrophic references (*z* = 2.01, *P* = 0.547).

## Discussion

Within the population studied, coralloid rhizomes marked a coordinated shift in fungal partner and carbon source. Individuals with coralloid rhizomes associated predominantly with *Coprinellus* (Psathyrellaceae) and were strongly ^13^C enriched, whereas those without coralloid rhizomes associated mainly with rhizoctonia fungi and remained isotopically close to autotrophic references. This sharp within-species contrast extends previous evidence that coralloid rhizomes facilitate Psathyrellaceae symbioses and greater fungal dependence, whereas rhizoctonia associations predominate in individuals lacking coralloid rhizomes (Yagame et al. 2021; Suetsugu et al. 2022; Zahn et al. 2022).

The Psathyrellaceae-associated state provided strong evidence of substantial fungal carbon acquisition by photosynthetic *C. appendiculata* individuals. Protocorms, coralloid rhizomes, and leaves of individuals bearing coralloid rhizomes were all strongly enriched in ^13^C relative to autotrophic reference plants. Psathyrellaceae symbionts, including extraradical rhizomorphs and pelotons at both stages of digestion, also consistently exhibited high ^13^C enrichment (ε^13^C = 7.3–8.7‰). This enrichment was lower than that reported for pelotons from fully mycoheterotrophic orchids exploiting wood-decaying fungi (ε^13^C = ca. 12–13‰), but higher than that reported for pelotons from mycoheterotrophic orchids exploiting ectomycorrhizal fungi (ε^13^C = 4.3–6.8‰) (Gomes et al. 2023; Zahn et al. 2023; Suetsugu and Okada 2025a; Suetsugu et al. 2025). Importantly, neither δ^13^C nor δ^15^N differed detectably between weakly and strongly digested pelotons. Peloton δ^15^N values also resembled those of extraradical rhizomorphs, but peloton δ^13^C values were significantly higher by approximately 1.3–1.4‰. Thus, we found no detectable digestion-stage effect, but pelotons and rhizomorphs should not be treated as interchangeable carbon isotope baselines. These conclusions are limited to the Psathyrellaceae-associated state of *C. appendiculata*.

Leaves of *C. appendiculata* individuals bearing coralloid rhizomes were also enriched in ^13^C (ε^13^C = 5.0‰), although less strongly than coralloid rhizomes, protocorms, and fungal tissues. Using protocorms or coralloid rhizome pelotons as operational endpoints yielded estimated fungal contributions of 50–60% to leaf carbon, consistent with substantial partial mycoheterotrophy. The rhizoctonia-associated state exhibited a contrasting isotopic pattern. Leaves of individuals lacking coralloid rhizomes showed little ^13^C enrichment (ε^13^C = 0.6‰) relative to autotrophic reference plants, consistent with predominantly autotrophic nutrition. Nevertheless, the ^13^C enrichment of rhizoctonia-associated pelotons (ε^13^C = 1.9‰) was low and overlapped with values observed in autotrophic reference plants. The mixing-model estimate for the rhizoctonia-associated state was therefore highly uncertain. Additional isotope markers, particularly δ^2^H, and preferably tracer experiments will be needed to detect or quantify small contributions of fungal carbon in the rhizoctonia-associated state (Zahn et al. 2022; Selosse et al. 2025).

The δ^15^N relationships between leaves and fungal tissues further illuminate nitrogen dynamics. Leaf δ^15^N values were significantly higher than those of the associated fungal tissues, particularly in the Psathyrellaceae-associated state. In this state, differences of 5.8–6.3‰ exceeded mean enrichments of 2.2–3.4‰ often reported between consumers and their resources (Minagawa and Wada 1984; Vander Zanden and Rasmussen 2001; McCutchan et al. 2003). Because Psathyrellaceae rhizomorphs and pelotons exhibited similar δ^15^N values regardless of digestion stage, this large difference is unlikely to result solely from ^15^N depletion during peloton degradation. The relatively small δ^15^N difference between coralloid rhizomes and fungal tissues suggests that selective transfer of isotopically distinct nitrogen compounds alone is unlikely to explain the full leaf–fungus difference. Therefore, the leaf–fungus difference likely reflects fractionation within the plant during nitrogen uptake, transfer, assimilation, and allocation to leaves. Similar leaf-to-root δ^15^N differences of 5–10‰ have been reported in partially mycoheterotrophic *Epipactis* species (Gonneau et al. 2014), whereas autotrophic plants generally exhibit much smaller isotopic differences between leaves and roots (Dijkstra et al. 2003).

Leaf nitrogen concentrations were also highest in individuals bearing coralloid rhizomes and exceeded those measured in coralloid rhizomes, protocorms, and pelotons. This pattern is consistent with preferential nitrogen allocation to leaves. Isotopic fractionation during nitrogen assimilation and allocation may also contribute to elevated leaf δ^15^N. Leaves are major sites of nitrogen assimilation, allocation, and temporary storage (Tegeder and Masclaux-Daubresse 2018), and partially mycoheterotrophic orchids often exhibit high leaf nitrogen concentrations and δ^15^N values similar to those of fully mycoheterotrophic species (Hynson et al. 2016). The present findings therefore suggest that large δ^15^N differences between leaves and fungal tissues may represent a physiological feature of at least some partially mycoheterotrophic orchids.

The fungal isotope patterns also raise the possibility that the orchid supplies some carbon to its fungi. Psathyrellaceae tissues associated with *C. appendiculata* were strongly enriched in ^13^C (ε^13^C = 7.3–8.7‰), but this enrichment was lower than that reported for pelotons from fully mycoheterotrophic orchids exploiting wood-decaying fungi (ε^13^C = ca. 12–13‰; Suetsugu et al. 2025; Suetsugu and Okada 2025a). In addition, Psathyrellaceae rhizomorphs intertwined with *C. appendiculata* coralloid rhizomes exhibited ^13^C enrichment (ε^13^C = 7.3‰) similar to that of the surrounding dead wood (ε^13^C = 6.4‰), whereas Kohzu et al. (1999) reported that fruiting bodies of wood-decaying fungi had δ^13^C values 3.1 ± 1.0‰ higher than those of the associated decomposed wood. One possible explanation is that ^13^C-depleted photosynthate from green *C. appendiculata* individuals contributes to the fungal carbon pool, thereby reducing the apparent ^13^C enrichment of the associated Psathyrellaceae fungi. Similarly, although the low ^13^C enrichment of rhizoctonia fungi may reflect endophytic nutrition in some lineages (Selosse and Martos 2014; Weiß et al. 2016), it may also partly reflect their acquisition of orchid-derived photosynthate.

Recent transcriptomic and metabolomic evidence further suggests that bidirectional carbon flow may persist in orchid mycorrhizas even under strongly heterotrophic conditions (Suetsugu et al. 2017; Zhao et al. 2025; Wang et al. 2026). Bidirectional carbon transfer has been demonstrated between the rhizoctonia-associated orchid *Goodyera repens* and its fungal partner (Cameron et al. 2006, 2008), and similar reciprocal exchange has been proposed for other rhizoctonia-associated orchids and some Pyroleae species (Lallemand et al. 2017; Selosse et al. 2025). Reciprocal carbon exchange may therefore occur broadly across orchid mycorrhizas, with trophic mode primarily determining the relative magnitudes and net balance of the opposing fluxes. However, some Psathyrellaceae species or strains may also differ intrinsically in substrate use or metabolic isotope fractionation (Han et al. 2026). Controlled culture experiments and reciprocal tracer labeling are therefore required to quantify carbon movement between *C. appendiculata* and its Psathyrellaceae partners.

Protocorms showed a related pattern. Their strong ^13^C enrichment (8.9 ± 1.8‰) confirmed dependence on fungal carbon but was slightly below the mean reported for fully mycoheterotrophic orchids exploiting wood-decaying fungi and below that of the predominantly leafless sister species *C. aphylla* (11–15‰; Suetsugu et al. 2022, 2026). Similarly, relatively low ^13^C enrichment has been reported for protocorms of *C. appendiculata* in Taiwan (8.0 ± 0.6‰) and *Oreorchis patens* (6.7 ± 0.2‰), both of which associate with Psathyrellaceae fungi (Zahn et al. 2022; Suetsugu and Okada 2025b). One hypothesis is that these patterns reflect partial inputs of ^13^C-depleted photosynthate from nearby conspecific green orchids through shared fungal networks. Such transfer has been demonstrated in the rhizoctonia-associated orchid *Dactylorhiza fuchsii* (Read et al. 2024), but was not tested here. Differences in fungal substrate use or isotope fractionation remain alternative explanations. Culture experiments comparing fungi grown with and without connections to adult plants would test whether adult-derived photosynthate alters the δ^13^C values of Psathyrellaceae fungi and their associated protocorms.

In conclusion, our integrated isotopic and metabarcoding analyses detected no significant difference in δ^13^C or δ^15^N between peloton digestion stages in *C. appendiculata* associated with Psathyrellaceae fungi. Pelotons and rhizomorphs had similar δ^15^N values but differed in δ^13^C, indicating that carbon isotope baselines depend on the fungal tissue sampled. The data also support pronounced nutritional plasticity within *C. appendiculata*, although studies of other populations are needed to determine how widespread these patterns are. Finally, the comparatively low ^13^C enrichment of Psathyrellaceae tissues and protocorms raises the possibility that some plant-derived carbon may be returned to fungal carbon pools, even when the orchid depends strongly on fungal carbon. Determining whether carbon moves in both directions between the orchid and its mycorrhizal fungi will require controlled culture experiments and tracer-based analyses.

## Supplementary Information

Below is the link to the electronic supplementary material.

Supplementary Material 1

## Data Availability

Sequence data have been deposited in the NCBI Sequence Read Archive under BioProject accession PRJNA1443979. Additional supporting information is available online in the Supporting Information accompanying this article.

## References

[CR1] Altschul SF, Madden TL, Schäffer AA, Zhang J, Zhang Z, Miller W, Lipman DJ (1997) Gapped BLAST and PSI-BLAST: a new generation of protein database search programs. Nucleic Acids Res 25:3389–3402. 10.1093/nar/25.17.3389 PMC146917 9254694

[CR2] Bates D, Mächler M, Bolker B, Walker S (2015) Fitting linear mixed-effects models using *lme4*. J Stat Softw 67:1–48. 10.18637/jss.v067.i01

[CR3] Bidartondo MI, Burghardt B, Gebauer G, Bruns TD, Read DJ (2004) Changing partners in the dark: isotopic and molecular evidence of ectomycorrhizal liaisons between forest orchids and trees. Proc R Soc B Biol Sci 271:1799–1806. 10.1098/rspb.2004.2807 PMC1691795 15315895

[CR4] Cameron DD, Leake JR, Read DJ (2006) Mutualistic mycorrhiza in orchids: evidence from plant–fungus carbon and nitrogen transfers in the green-leaved terrestrial orchid *Goodyera repens*. New Phytol 171:405–416. 10.1111/j.1469-8137.2006.01767.x 16866946

[CR5] Cameron DD, Johnson I, Read DJ, Leake JR (2008) Giving and receiving: measuring the carbon cost of mycorrhizas in the green orchid *Goodyera repens*. New Phytol 180:176–184. 10.1111/j.1469-8137.2008.02533.x 18627489

[CR6] Dearnaley JDW, Martos F, Selosse M-A (2012) Orchid mycorrhizas: molecular ecology, physiology, evolution and conservation aspects. In: Hock B (ed) Fungal associations. The Mycota. Springer, Berlin, pp 207–230. 10.1007/978-3-642-30826-0_12

[CR7] Dijkstra P, Williamson C, Menyailo O, Doucett R, Koch G, Hungate BA (2003) Nitrogen stable isotope composition of leaves and roots of plants growing in a forest and a meadow. Isot Environ Health Stud 39:29–39. 10.1080/1025601031000102189 12812253

[CR8] Doyle JJ, Doyle JL (1990) Isolation of plant DNA from fresh tissue. Focus 12:13–15

[CR9] Esling P, Lejzerowicz F, Pawlowski J (2015) Accurate multiplexing and filtering for high-throughput amplicon sequencing. Nucleic Acids Res 43:2513–2524. 10.1093/nar/gkv107 PMC4357712 25690897

[CR10] Gebauer G, Meyer M (2003) ^15^N and ^13^C natural abundance of autotrophic and mycoheterotrophic orchids provides insight into nitrogen and carbon gain from fungal association. New Phytol 160:209–223. 10.1046/j.1469-8137.2003.00872.x 33873535

[CR11] Gebauer G, Preiss K, Gebauer AC (2016) Partial mycoheterotrophy is more widespread among orchids than previously assumed. New Phytol 211:11–15. 10.1111/nph.13865 26832994

[CR12] Gomes SIF, Giesemann P, Klink S, Hunt C, Suetsugu K, Gebauer G (2023) Stable isotope natural abundances of fungal hyphae extracted from the roots of arbuscular mycorrhizal mycoheterotrophs and rhizoctonia-associated orchids. New Phytol 239:1166–1172. 10.1111/nph.18990 37343595

[CR13] Gonneau C, Jersáková J, de Tredern E, Till-Bottraud I, Saarinen K, Sauve M, Roy M, Hájek T, Selosse M-A (2014) Photosynthesis in perennial mixotrophic *Epipactis* spp. (Orchidaceae) contributes more to shoot and fruit biomass than to hypogeous survival. J Ecol 102:1183–1194. 10.1111/1365-2745.12274

[CR14] Govaerts R (2026) The World Checklist of Vascular Plants (WCVP). Royal Botanic Gardens, Kew. Checklist dataset. 10.15468/6h8ucr. Accessed 29 August 2026

[CR15] Guindon S, Dufayard J-F, Lefort V, Anisimova M, Hordijk W, Gascuel O (2010) New algorithms and methods to estimate maximum-likelihood phylogenies: assessing the performance of PhyML 3.0. Syst Biol 59:307–321. 10.1093/sysbio/syq010 20525638

[CR16] Han Z, Zahn FE, Jiang H, Lee Y-I, Gebauer G (2026) Stable isotope signatures and specific mycobionts reveal the mycoheterotrophic nutrient acquisition of *Danxiaorchis yangii*. Mycorrhiza 36:44. 10.1007/s00572-026-01288-x 42384172

[CR17] Hoang DT, Chernomor O, von Haeseler A, Minh BQ, Vinh LS (2018) UFBoot2: improving the ultrafast bootstrap approximation. Mol Biol Evol 35:518–522. 10.1093/molbev/msx281 PMC5850222 29077904

[CR18] Hobbie EA, Colpaert JV (2003) Nitrogen availability and colonization by mycorrhizal fungi correlate with nitrogen isotope patterns in plants. New Phytol 157:115–126. 10.1046/j.1469-8137.2003.00657.x 33873704

[CR19] Hothorn T, Bretz F, Westfall P (2008) Simultaneous inference in general parametric models. Biom J 50:346–363. 10.1002/bimj.200810425 18481363

[CR20] Huson DH, Auch AF, Qi J, Schuster SC (2007) MEGAN analysis of metagenomic data. Genome Res 17:377–386. 10.1101/gr.5969107 PMC1800929 17255551

[CR21] Hynson NA, Madsen TP, Selosse M-A, Adam IKU, Ogura-Tsujita Y, Roy M, Gebauer G (2013) The physiological ecology of mycoheterotrophy. In: Merckx VSFT (ed) Mycoheterotrophy: the biology of plants living on fungi. Springer, New York, pp 297–342. 10.1007/978-1-4614-5209-6_8

[CR22] Hynson NA, Schiebold JMI, Gebauer G (2016) Plant family identity distinguishes patterns of carbon and nitrogen stable isotope abundance and nitrogen concentration in mycoheterotrophic plants associated with ectomycorrhizal fungi. Ann Bot 118:467–479. 10.1093/aob/mcw119 PMC4998980 27451987

[CR23] Jacquemyn H, Merckx VSFT (2019) Mycorrhizal symbioses and the evolution of trophic modes in plants. J Ecol 107:1567–1581. 10.1111/1365-2745.13165

[CR24] Julou T, Burghardt B, Gebauer G, Berveiller D, Damesin C, Selosse M-A (2005) Mixotrophy in orchids: insights from a comparative study of green individuals and nonphotosynthetic individuals of *Cephalanthera damasonium*. New Phytol 166:639–653. 10.1111/j.1469-8137.2005.01364.x 15819926

[CR25] Kalyaanamoorthy S, Minh BQ, Wong TKF, von Haeseler A, Jermiin LS (2017) ModelFinder: fast model selection for accurate phylogenetic estimates. Nat Methods 14:587–589. 10.1038/nmeth.4285 PMC5453245 28481363

[CR26] Kohzu A, Yoshioka T, Ando T, Takahashi M, Koba K, Wada E (1999) Natural ^13^C and ^15^N abundance of field-collected fungi and their ecological implications. New Phytol 144:323–330. 10.1046/j.1469-8137.1999.00508.x

[CR27] Lallemand F, Puttsepp Ü, Lang M, Luud A, Courty P-E, Palancade C, Selosse M-A (2017) Mixotrophy in Pyroleae (Ericaceae) from Estonian boreal forests does not vary with light or tissue age. Ann Bot 120:361–371. 10.1093/aob/mcx054 PMC5591414 28575199

[CR28] Lee Y-I, Yang C-K, Gebauer G (2015) The importance of associations with saprotrophic non-rhizoctonia fungi among fully mycoheterotrophic orchids is currently underestimated: novel evidence from subtropical Asia. Ann Bot 116:423–435. 10.1093/aob/mcv085 PMC4549957 26113634

[CR29] Löytynoja A (2021) Phylogeny-aware alignment with PRANK and PAGAN. In: Katoh K (ed) Multiple sequence alignment: methods and protocols. Springer, New York, pp 17–37. 10.1007/978-1-0716-1036-7_2 33289884

[CR30] Martos F, Dulormne M, Pailler T, Bonfante P, Faccio A, Fournel J, Dubois M-P, Selosse M-A (2009) Independent recruitment of saprotrophic fungi as mycorrhizal partners by tropical achlorophyllous orchids. New Phytol 184:668–681. 10.1111/j.1469-8137.2009.02987.x 19694964

[CR31] McCutchan JH Jr, Lewis WM Jr, Kendall C, McGrath CC (2003) Variation in trophic shift for stable isotope ratios of carbon, nitrogen, and sulfur. Oikos 102:378–390. 10.1034/j.1600-0706.2003.12098.x

[CR32] Merckx VSFT (ed) (2013) Mycoheterotrophy: the biology of plants living on fungi. Springer, New York. 10.1007/978-1-4614-5209-6

[CR33] Minagawa M, Wada E (1984) Stepwise enrichment of ^15^N along food chains: further evidence and the relation between δ^15^N and animal age. Geochim Cosmochim Acta 48:1135–1140. 10.1016/0016-7037(84)90204-7

[CR34] Nilsson RH, Larsson K-H, Taylor AFS, Bengtsson-Palme J, Jeppesen TS, Schigel D, Kennedy P, Picard K, Glöckner FO, Tedersoo L et al (2019) The UNITE database for molecular identification of fungi: handling dark taxa and parallel taxonomic classifications. Nucleic Acids Res 47:D259–D264. 10.1093/nar/gky1022 PMC6324048 30371820

[CR35] Ogura-Tsujita Y, Gebauer G, Hashimoto T, Umata H, Yukawa T (2009) Evidence for novel and specialized mycorrhizal parasitism: the orchid *Gastrodia confusa* gains carbon from saprotrophic *Mycena*. Proc R Soc B Biol Sci 276:761–767. 10.1098/rspb.2008.1225 PMC2660934 19004757

[CR36] Ogura-Tsujita Y, Yukawa T, Kinoshita A (2021) Evolutionary histories and mycorrhizal associations of mycoheterotrophic plants dependent on saprotrophic fungi. J Plant Res 134:19–41. 10.1007/s10265-020-01244-6 PMC7817554 33417080

[CR37] Oja J, Kohout P, Tedersoo L, Kull T, Kõljalg U (2015) Temporal patterns of orchid mycorrhizal fungi in meadows and forests as revealed by 454 pyrosequencing. New Phytol 205:1608–1618. 10.1111/nph.13223 25546739

[CR38] Preiss K, Gebauer G (2008) A methodological approach to improve estimates of nutrient gains by partially mycoheterotrophic plants. Isot Environ Health Stud 44:393–401. 10.1080/10256010802507458 19061069

[CR39] R Core Team (2026) R: a language and environment for statistical computing. R Foundation for Statistical Computing, Vienna, Austria. https://www.R-project.org/

[CR40] Rammitsu K, Kajita T, Imai R, Ogura-Tsujita Y (2021) Strong primer bias for Tulasnellaceae fungi in metabarcoding: specific primers improve the characterization of the mycorrhizal communities of epiphytic orchids. Mycoscience 62:356–363. 10.47371/mycosci.2021.06.005 PMC9721509 37090180

[CR41] Rasmussen HN (2002) Recent developments in the study of orchid mycorrhiza. Plant Soil 244:149–163. 10.1023/A:1020246715436

[CR42] Read DJ, Haggar J, Magkourilou E, Durant E, Johnson D, Leake JR, Field KJ (2024) Photosynthate transfer from an autotrophic orchid to conspecific heterotrophic protocorms through a common mycorrhizal network. New Phytol 243:398–406. 10.1111/nph.19810 38757767

[CR43] Rognes T, Flouri T, Nichols B, Quince C, Mahé F (2016) VSEARCH: a versatile open source tool for metagenomics. PeerJ 4:e2584. 10.7717/peerj.2584 PMC5075697 27781170

[CR44] Selosse M-A, Martos F (2014) Do chlorophyllous orchids heterotrophically use mycorrhizal fungal carbon? Trends Plant Sci 19:683–685. 10.1016/j.tplants.2014.09.005 25278267

[CR45] Selosse M-A, Alaux P-L, Deloche L, Delannoy E, Minasiewicz J, Tsiftsis S, Figura T, Martos F (2025) Mixotrophy in orchids: facts, questions, and perspectives. New Phytol 246:1912–1921. 10.1111/nph.70106 PMC12059541 40195594

[CR50] Suetsugu K, Okada H (2025a) Integrated peloton and fruiting body isotope data shed light on mycoheterotrophic interactions in *Gastrodia pubilabiata* (Orchidaceae). Mycorrhiza 35:43. 10.1007/s00572-025-01213-8 PMC12158851 40498228

[CR51] Suetsugu K, Okada H (2025b) Subterranean morphology underpins the degree of mycoheterotrophy, mycorrhizal associations, and plant vigor in a green orchid *Oreorchis patens*. Plant J 121:e70045. 10.1111/tpj.70045 PMC11837900 39969432

[CR46] Suetsugu K, Yamato M, Miura C, Yamaguchi K, Takahashi K, Ida Y, Shigenobu S, Kaminaka H (2017) Comparison of green and albino individuals of the partially mycoheterotrophic orchid *Epipactis helleborine* on molecular identities of mycorrhizal fungi, nutritional modes, and gene expression in mycorrhizal roots. Mol Ecol 26:1652–1669. 10.1111/mec.14021 28099773

[CR47] Suetsugu K, Matsubayashi J, Tayasu I (2020) Some mycoheterotrophic orchids depend on carbon from dead wood: novel evidence from a radiocarbon approach. New Phytol 227:1519–1529. 10.1111/nph.16409 31985062

[CR48] Suetsugu K, Haraguchi TF, Tayasu I (2022) Novel mycorrhizal cheating in a green orchid: *Cremastra appendiculata* depends on carbon from deadwood through fungal associations. New Phytol 235:333–343. 10.1111/nph.17313 33822388

[CR49] Suetsugu K, Matsubayashi J, Okada H (2025) Stable isotope signatures illuminate diverse nutritional strategies in rhizoctonias and their orchid partners. Plant Cell Environ 48:792–804. 10.1111/pce.15167 39351829

[CR52] Suetsugu K, Okada H, Hirota SK, Suyama Y (2026) Isotopic and genetic evidence of partial mycoheterotrophy in leafy variants of *Cremastra aphylla*, a predominantly leafless orchid. Ann Bot 137:689–701. 10.1093/aob/mcaf235 PMC12933686 40996154

[CR53] Syed F, Grunenwald H, Caruccio N (2009) Optimized library preparation method for next-generation sequencing. Nat Methods 6:i–ii. 10.1038/nmeth.f.269

[CR54] Tanabe AS, Toju H (2013) Two new computational methods for universal DNA barcoding: a benchmark using barcode sequences of bacteria, archaea, animals, fungi, and land plants. PLoS ONE 8:e76910. 10.1371/journal.pone.0076910 PMC3799923 24204702

[CR56] Tayasu I, Hirasawa R, Ogawa NO, Ohkouchi N, Yamada K (2011) New organic reference materials for carbon- and nitrogen-stable isotope ratio measurements provided by Center for Ecological Research, Kyoto University, and Institute of Biogeosciences, Japan Agency for Marine-Earth Science and Technology. Limnology 12:261–266. 10.1007/s10201-011-0345-5

[CR55] Taylor AFS, Högbom L, Högberg M, Lyon AJE, Näsholm T, Högberg P (1997) Natural ^15^N abundance in fruit bodies of ectomycorrhizal fungi from boreal forests. New Phytol 136:713–720. 10.1046/j.1469-8137.1997.00788.x 33863102

[CR57] Tegeder M, Masclaux-Daubresse C (2018) Source and sink mechanisms of nitrogen transport and use. New Phytol 217:35–53. 10.1111/nph.14876 29120059

[CR58] Trudell SA, Rygiewicz PT, Edmonds RL (2003) Nitrogen and carbon stable isotope abundances support the mycoheterotrophic nature and host specificity of certain achlorophyllous plants. New Phytol 160:391–401. 10.1046/j.1469-8137.2003.00876.x 33832180

[CR59] Vander Zanden MJ, Rasmussen JB (2001) Variation in δ^15^N and δ^13^C trophic fractionation: implications for aquatic food web studies. Limnol Oceanogr 46:2061–2066. 10.4319/lo.2001.46.8.2061

[CR60] Veldre V, Abarenkov K, Bahram M, Martos F, Selosse M-A, Tamm H, Kõljalg U, Tedersoo L (2013) Evolution of nutritional modes of Ceratobasidiaceae (Cantharellales, Basidiomycota) as revealed from publicly available ITS sequences. Fungal Ecol 6:256–268. 10.1016/j.funeco.2013.03.004

[CR61] Wang Y, Zhao Z, Liu J, Yang A, Jacquemyn H, Yang L, Qian X, Li T, Ding G, Xing X (2026) Contribution of orchids to the carbon budget of fungi in germinating seeds of *Gymnadenia conopsea*. Sci China Life Sci 69:662–675. 10.1007/s11427-025-2987-9 40762940

[CR62] Waud M, Busschaert P, Ruyters S, Jacquemyn H, Lievens B (2014) Impact of primer choice on characterization of orchid mycorrhizal communities using 454 pyrosequencing. Mol Ecol Resour 14:679–699. 10.1111/1755-0998.12229 24460947

[CR63] Weiß M, Waller F, Zuccaro A, Selosse M-A (2016) Sebacinales – one thousand and one interactions with land plants. New Phytol 211:20–40. 10.1111/nph.13977 27193559

[CR64] Wong TKF, Ly-Trong N, Ren H, Demotte P, Baños H, Roger AJ, Susko E, Bielow C, De Maio N, Goldman N et al (2026) IQ-TREE 3: phylogenomic inference software using complex evolutionary models. Mol Biol Evol 43:msag117. 10.1093/molbev/msag117 PMC13191116 42085559

[CR65] Yagame T, Lallemand F, Selosse M-A, Funabiki E, Yukawa T (2021) Mycobiont diversity and first evidence of mixotrophy associated with Psathyrellaceae fungi in the chlorophyllous orchid *Cremastra variabilis*. J Plant Res 134:1213–1224. 10.1007/s10265-021-01337-w 34405353

[CR66] Zahn FE, Lee Y-I, Gebauer G (2022) Fungal association and root morphology shift stepwise during ontogenesis of the orchid *Cremastra appendiculata* towards autotrophic nutrition. AoB Plants 14:plac021. 10.1093/aobpla/plac021 PMC9167560 35673361

[CR67] Zahn FE, Söll E, Chapin TK, Wang D, Gomes SIF, Hynson NA, Pausch J, Gebauer G (2023) Novel insights into orchid mycorrhiza functioning from stable isotope signatures of fungal pelotons. New Phytol 239:1449–1463. 10.1111/nph.18991 37343598

[CR68] Zhao Z, Wang Y, Yang L, Qian X, Yang A, Liu J, Jacquemyn H, Li T, Xing X (2025) Metabolic shifts and nutrient transfer patterns in orchid seeds during symbiotic germination. Plant Cell Environ 48:6406–6420. 10.1111/pce.15612 40369997

